# Acute glomerulonephritis in a hematopoietic blood stem cell donor 

**DOI:** 10.5414/CNCS110538

**Published:** 2021-07-01

**Authors:** Preeti Chandra, Saurabh Dahiya, Gabriela Sanchez-Petitto, Jawad Malik, Jonathan Bolanos, Abdolreza Haririan, Matthew Weir, Cinthia Drachenberg, Aaron Rapoport

**Affiliations:** 1 Division of Nephrology,; 2 Division of Hematology Oncology, and; 3 Department of Pathology, University of Maryland, Baltimore, MD, USA

**Keywords:** acute pauci-immune glomerulonephritis, ANCA-positive glomerulonephritis, stem cell donor, granulocyte colony-stimulating factor

## Abstract

Use of granulocyte colony-stimulating factor (G-CSF) has been associated with side effects including reports of acute glomerulonephritis (GN), almost all of which have been immune complex associated. There is one prior report of pauci-immune GN in a child, but was negative for ANCA (anti-neutrophilic cytoplasmic antibodies). We describe the first case of ANCA-positive pauci-immune GN exacerbated by the use of G-CSF for peripheral blood stem cell (PBSC) donation in a patient with no prior history of vasculitis. Given the use of G-CSF in PBSC donation and neutropenias associated with various conditions, it is important that both the nephrologist and the hematologist are aware of the renal risks associated with its use.

## Introduction 

Granulocyte colony-stimulating factor (G-CSF) is a hematopoietic cytokine produced by monocytes, fibroblasts, endothelial cells, and in normal humans it leads to increased production of polymorphonuclear (PMN) leucocytes as well as dose-dependent reduction in their maturation time in the bone marrow, acts as a powerful activator of neutrophils, and enhances the respiratory burst in PMNs in response to various stimuli [1]. 

We describe a patient who developed acute glomerulonephritis after being given G-CSF for stem cell mobilization as part of stem cell donation to his father. 

## Case presentation 

The patient is a 30-year-old Caucasian male with no past medical history who was evaluated as a stem cell donor for his father. The father had T-cell prolymphocytic leukemia and was undergoing treatment with allogenic stem cell transplant. The patient was otherwise healthy. He worked as a personal trainer and denied any use of recreational drugs or over-the-counter medications including use of non-steroidal anti-inflammatory drugs. He worked out almost daily at the gym and ate a high-protein diet comprising 200 grams of dietary protein which was mostly meat based (2 g/kg body weight for his weight of 100 kg) and took ~ 5 g daily of creatine supplement. On initial assessment by the bone marrow transplant (BMT) team, the patient stated that he felt well and was asymptomatic. He had a creatinine of 1.86 mg/dL) and a urinalysis showed 2+ blood and 1+ protein, with 3 – 5 RBC/hpf. He was counseled to reduce the meat protein in his diet, stop the creatine supplement, and hold his exercise workouts. Repeat labs a week later showed a creatinine of 1.45 mg/dL. A simultaneous 24-hour urine collection done at this time showed a creatinine clearance of 101 mL/min for his body surface area of 2.35 m^2^. The creatinine clearance was 74 mL/min/1.73m^2^. 

Given improvement in his creatinine with decrease of his protein intake and the window of opportunity to donate stem cells to his father, the BMT team decided to proceed with stem cell donation. He was started on 10 µg/kg/day dose of granulocyte-G-CSF (Granix) to be taken for a total of 5 days. Within a day of starting G-CSF, the patient developed bone pain, and over the subsequent days developed sore throat and some left upper abdominal discomfort, all anticipated side effects. He also noticed dark tea-colored urine after receiving 3 doses of G-CSF. He completed the duration of the G-CSF treatment and had repeat labs and urinalysis done on the day of apheresis and stem cell collection. Urinalysis showed 3+ blood, 1+ protein, more than 50 RBC/hpf, and serum creatinine was up to 2 mg/dL, though a repeat lab later in the afternoon showed creatinine of 1.8 mg/dL, serum total leucocyte count was 43,400/µL (69% neutrophils). The patient was asked to hydrate. Labs were repeated on the 2^nd^ day after donation and showed creatinine of 2.3 mg/dL, urinalysis showed 3+ blood, 1+ protein, and > 50 RBC/hpf. He was admitted to the hospital, and nephrology was consulted. His vital signs were normal. His exam was unremarkable except some discomfort over the left upper quadrant. He had slightly tea-colored urine. Urine microscopy showed ~ 25 to 30 RBC/hpf ~ 25% acanthocytes, and there were no RBC casts. He had a baseline creatinine of 1.3 mg/dL ~ 2 years prior to presentation, but no other prior renal function tests, including urinalysis, were available. Platelets were decreased on admission, 83,000 cells/µL, from a normal baseline and dropped further to a nadir of 62,000 cells/µL the next day. This was attributed to the use of G-CSF and the apheresis procedure. His serum creatinine peaked at 2.45 mg/dL and then stabilized in the 2 – 2.2 mg/dL range. In subsequent days, he felt his urine color became pale yellow. A repeat urinalysis showed 11 – 25 RBC/hpf, and a spot urine showed 0.4 g per g of creatinine and albumin/creatinine ratio 0.2 g/g. 

**Figure 1:**
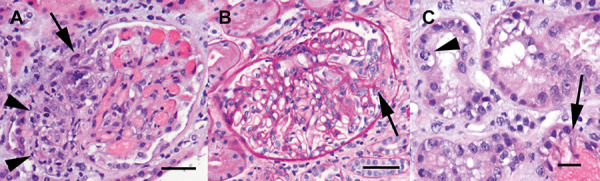
Renal biopsy. A: Segmental, necrotizing lesion with formation of an epithelial crescent (arrow). The adjacent glomerular arteriole shows evidence of vasculitis consisting of mural inflammation, karyorrhexis, and luminal obliteration (arrowheads). B: Segmental area of glomerular rarefaction with a small fibroepithelial crescent and adhesion to the Bowman’s capsule (arrow). Typical of a pauci-immune crescentic process, the remaining glomerular tufts are normocellular and have patent capillary lumina. C: Acute tubular injury with a tubular cell mitosis (arrowhead) and red cell casts were also noted (arrow). Bars: 20 µm.

When platelets improved to 85,000 cells/µL, he underwent a renal biopsy (Figure 1). This showed 13 glomeruli, of which none were globally sclerosed. Three glomeruli showed small segmental areas of tuft solidification and/or fibrinoid necrosis in association with epithelial crescents. The remaining tufts were open, capillary lumina appeared normocellular, with normal peripheral capillary basement membranes and no thrombotic changes. There was minimal interstitial fibrosis with no significant evidence of tubular atrophy (< 5% of the cortical areas sampled). The tubules showed acute tubular injury consisting of cytoplasmic swelling and vacuolization, apical blebbing, and occasional mitosis. There were clusters of tubules with red cell casts. Interlobular arteries showed mild wall thickening with no significant sclerosis. Congo red staining for amyloid was negative. Immunofluorescence on 2 – 3 glomeruli (1 with a crescent) was negative for IgG, IgM, IgA, C3, C1q, and κ and λ light chains. Electron microscopy showed 1 glomerulus with a minute area of parietal podocyte hyperplasia. Definite necrotizing and crescentic changes were not present. Overall, there was preserved glomerular architecture with mild accentuation of the mesangial areas. The peripheral capillary basement membranes were overall normal in thickness and texture. Podocyte foot processes were well formed. No immune-type electron-dense deposits were seen. No fibrils, tubuloreticular inclusions, or amyloid were present. The final diagnosis was focal necrotizing and crescentic glomerulonephritis, pauci-immune, no significant interstitial fibrosis and tubular atrophy. 

Serologies showed positive anti-nuclear antibody at a titer of 1 : 160 (reference range < 1 : 80), normal complement factors C3 and C4, and negative double-stranded DNA antibody. Additional tests that returned after the renal biopsy were a positive titer (1 : 160, reference range < 1 : 20) for anti-neutrophilic cytoplasmic antibodies (ANCA) in a peri-nuclear pattern on immunofluorescence (p-ANCA). Myeloperoxidase (MPO) antibody was detected, with a level of 74 AU/mL (reference range 0 – 19 AU/mL). Antibodies against proteinase 3 (PR3) and glomerular basement membrane were absent. 

He was started on high-dose glucocorticoid after the biopsy and received pulse methyprednisolone 1 g daily for 2 consecutive days followed by high-dose prednisone at 80 mg daily, which was subsequently decreased to 60 mg daily. He was also given rituximab (1 g) and started on prophylaxis for *Pneumocystis carinii* infection. He subjectively felt better, the urine became clearer, and he was subsequently discharged. He received the second dose of rituximab 1 g 2 weeks after the first dose. We selected a 1-g rituximab dose for 2 doses (as opposed to 375 mg/m^2^ weekly doses for 4 doses) given the current COVID-19 pandemic. 0% lymphocytes in the blood were positive for CD19/20 after second rituximab dose. The prednisone dose has been slowly decreased outpatient. 

At last evaluation ~ 10 weeks after the start of glucocorticoids and rituximab, his creatinine is ranging between 1.4 and 1.5 mg/dL, urinalysis shows microscopic hematuria, and the MPO titer is lower. Spot urine protein/creatinine ratio is 0.3 – 0.4 g/g, and a 24-hour collection shows urine protein of 1.5 g/day. 

## Discussion 

The administration of recombinant human G-CSF (rhG-CSF) to normal healthy individuals stimulates proliferation of myeloid precursors, increases the neutrophil release from bone marrow, causes activation of neutrophils including increased respiratory burst, expression of surface CD11b/CD18 antigens, and increased elastase activity [2, 3]. G-CSF also results in increased number as well as activation of monocytes, increased markers of endothelial activation and coagulation [2, 3]. For stem cell donation, an optimal dose of rhG-CSF is one that ensures collection of an adequate number of CD34 + progenitor cells while minimizing donor’s exposure to the side effects of the cytokine. A peak increase in the circulating CD34+ progenitor cells usually occurs after 4 – 6 days of G-CSF therapy at a dose of 10 μg/kg/day. A wide range of dosages (3 – 20 µg/kg/day) have been used for healthy peripheral blood stem cell (PBSC) donation with dose-response effect and increased risk of side effects with doses higher than 10 µg/kg/day [4, 5, 6]. PBSC donation can be associated with side effects including bone pain, fatigue, flu-like symptoms, myalgias, insomnia, nausea, dizziness, anorexia, thrombocytopenia, hypocalcemia, and spleen enlargement. Serious adverse events are uncommon but reported and include cardiovascular events, acute lung injury, thromboembolic events, bleeding, splenic rupture, activation of autoimmunity, seizures, association with hematological malignancy, though the incidence of the latter is not higher than the expected incidence in the age- and sex-adjusted general population and the exact association remains unclear [6, 7, 8, 9]. 

G-CSF has been associated with side effects including neutrophilic dermatosis and leucocytoclastic vasculitis [10]. In a case series of 18 patients with cutaneous vasculitis due to G-CSF [11], most were leucocytoclastic vasculitis which mostly occurred when absolute neutrophil count was above 800/µL. In this series, 3 patients were reported to have hematuria and proteinuria which resolved with the skin lesions; no renal biopsy was available. The use of G-CSF has been associated with reactivation of rheumatoid arthritis [12], as well as associated with flare or increased activity of the primary autoimmune disease prior to bone marrow transplant for such patients [13]. 

G-CSF has been associated with development of acute glomerulonephritis as documented in case reports summarized in Supplemental Table 1. While some of them resolved with holding G-CSF, in other cases, immunosuppressants were given in addition. In most cases, holding G-CSF +/- immunosuppressive therapy improved the clinical picture but in some cases, patients had irreversible renal injury. Almost all of these cases of acute glomerulonephritis were immune complex associated. There is one report of pauci-immune glomerulonephritis occurring in a child but it was ANCA negative (reference 15 in Supplemental Table 1). There have been no prior reports of ANCA-positive pauci-immune glomerulonephritis associated with use of G-CSF in a patient with no known history of vasculitis or autoimmune disease. 

In our case report, the patient developed acutely worsening renal function, hematuria, and proteinuria within days of receiving a standard-dose G-CSF. Renal biopsy was consistent with focal necrotizing crescentic glomerulonephritis with immunofluorescence showing a pauci-immune pattern. Subsequently, he tested positive for p-ANCA and anti-MPO antibodies consistent with microscopic polyangiitis. His baseline renal function was likely confounded to some degree by the high dietary intake of animal protein (~ 2 g/kg bodyweight) and to a small extent by the use of creatine supplements. This is evidenced by some improvement in his creatinine after he was asked to decrease the protein in his diet and hold creatine supplements. His baseline urinalysis showed small amount of microscopic hematuria, and baseline creatinine clearance was not normal which would indicate he likely had some baseline disease activity, which was undiagnosed. While there were no baseline levels of ANCA titers available prior to use of G-CSF, given his baseline urinalysis was abnormal, it is possible that he would have tested positive for the antibodies. However, he clearly had acute kidney injury and worsening hematuria consistent with an acute glomerulonephritis after use of G-CSF. Renal biopsy showed acute necrotizing lesions and acute tubular injury, there was minimal fibrosis consistent with the acute nature of the presentation. We cannot rule out a greater degree of fibrosis or chronicity that was missed on the renal biopsy. 

Hellmich et al. [14] reported that recombinant human granulocyte colony-stimulating factor (rHuG-CSF) could be safely used in 6 patients with granulomatosis with polyangiitis (previously Wegener’s granulomatosis), all PR3 antibody positive, to treat severe neutropenia (absolute neutrophil count < 1,000/µL) after being treated with cyclophosphamide without causing disease flare. Of note, in this study, rHuG-CSF was used only to correct the neutropenia and the drug was stopped when the neutrophil count was above 1,000/µL, the mean duration of use was 3.8 ± 0.8 days, and the patients had been on cyclophosphamide prior to the neutropenia that could have prevented a disease flare. 

However, in a report of 2 patients with known ANCA-associated vasculitis, both positive for PR3 antibody, there was a clinical flare of the disease after receiving G-CSF [15]. The patients were given G-CSF in preparation for autologous stem cell collection as rescue treatment for their autoimmune diseases. The first patient received a dose of intravenous cyclophosphamide followed by 300 µg of filgrastim daily for 8 days when the patient developed a disease flare that required high-dose steroids. The second patient received a dose of intravenous cyclophosphamide followed by a reduced dose of G-CSF 300 µg for 4 days as well prednisone 50 mg daily prophylactically. The patient still had a disease flare and required start of oral cyclophosphamide. The total leucocyte count was 35,400/µL for the first patient and 10,700/µL for the second patient when they had a flare [15]. 

Freeley et al. [16] noted that serum levels of G-CSF were significantly higher in patients with active ANCA vasculitis compared with age-matched healthy controls. In vitro priming of human neutrophils with G-CSF prior to administration of anti-MPO ANCA (but not anti-PR3 ANCA) resulted in greater respiratory burst consistent with greater neutrophil activation. They also noted that stimulation of neutrophils by G-CSF in murine model of anti-MPO crescentic glomerulonephritis resulted in greater histological severity of glomerulonephritis compared with controls. In vitro, G-CSF did not increase expression of MPO or PR3 on the neutrophil surface [16]. 

Hellmich et al. [17] describe that in in vitro studies of neutrophils from normal individuals, G-CSF did not significantly alter membrane PR3 expression on neutrophils that are either untreated or that are primed with tumor necrosis factor alpha (TNF-α). However, granulocyte monocyte colony-stimulating factor (GM-CSF) significantly increased PR3 expression on both intact and TNF-α-primed neutrophils. G-CSF or GM-CSF did not significantly alter MPO expression on the neutrophils [17]. 

In a mouse model of neutrophil-mediated heterologous-phase anti-glomerular basement membrane (GBM) glomerulonephritis, G-CSF knock-out mice were protected from anti-GBM glomerulonephritis compared with wild-type mice [18]. However, G-CSF knockout mice were not protected from T-cell/macrophage-mediated crescentic autologous-phase anti-GBM glomerulonephritis. This suggests that C-CSF has a role in neutrophil-mediated glomerular injury but not in experimental crescentic glomerulonephritis [18]. 

ANCA has been found in normal individuals, though in lower amounts compared to patients with vasculitis [19, 20], and thought to react to different epitopes compared with pathogenic ANCA [21]. Several lines of evidence suggest that ANCA antibodies are pathogenic in ANCA-associated vasculitis [22, 23, 24, 25]. Studies suggest that pathogenic ANCA react to the neutrophilic antigens (PR3, MPO) present on the surface of the neutrophils, resulting in production of reactive oxygen species as well as neutrophil extracellular traps (NETs), which contain chromatin material lined with proinflammatory proteins including MPO and PR3. NETs result in endothelial injury, activation of alternate complement pathway, and transfer of MPO and PR3 to the myeloid dendritic cells with resultant activation of autoreactive T and B cells and production of ANCA antibodies [22, 23, 24, 25]. 

Given the baseline abnormal urinalysis, it is plausible that our patient had underlying ANCA-associated disease that was undiagnosed. However, he clearly had an acute exacerbation of disease with use of G-CSF. Given the role of G-CSF in stimulating neutrophils and the role of neutrophils and ANCA antibodies in the pathogenesis of ANCA associated vasculitis (AAV), the possibility of developing or exacerbating an acute ANCA-associated pauci-immune glomerulonephritis with G-CSF should be considered. Consideration should be given for evaluating for undiagnosed AAV before administration of G-CSF and it should be added to the list of drugs that are associated with triggering AAV. 

## Funding 

No funding agency had a role in the preparation or review of this manuscript. 

## Conflict of interest 

None of the authors declare a competing interest or conflict of interest in this manuscript. 

## Supplemental material

Supplemental material Supplementary Data - Table 1
