# Supplementary material for: Acute glomerulonephritis in a hematopoietic blood stem cell donor
Source: Clin Nephrol Case Stud. 2021 Jul 1;9:81–6. doi: 10.5414/CNCS110538 (PMC8259466; doi:10.5414/CNCS110538)
Supplement: Supplemental material [file CNCS-9-081-01-S01.pdf]

Supplementary Data -Table 1

Number of patients	***Renal presentation including biopsy finding/Type of Glomerulonephritis***	***Indication for G-CSF***	***Comments***	***Study Reference***
1	Hematuria, proteinuria and renal dysfunction. Renal biopsy consistant with Mesangio proliferative GN	Severe congenital neutropenia	Long term use of G-CSF. Symptoms resolved after discontinuation of G-CSF	1
1 (child)	Hematuria, proteinuria, severe renal impairment. Renal biopsy consistant with crescentic GN c/w rapidly progressive GN. No glomerular immune deposits in biopsy. Negative anti glomerular basement membrane and negative ANCA antibodies.	Severe congenital neutropenia	On long term G-CSF. RPGN treated with steroids and cyclophosphamide with improvement in renal function, proteinuria persisted.	2
1 (child)	Microhematuria, recurrent macrohematuria, non nephrotic proteinuria for years. Then developed worsening renal dysfunction and proteinuria. Renal biopsy c/w MPGN. IF with IgM and C3 deposition	Severe congenital neutropenia	Renal function improved with holding G-CSF and steroids but worsened with resumption of G-CSF. Patient later positive for HCV but renal dysfunction/proteinuria despite HCV treatment and disappearance of HCV in blood. Authors noted improvement with change of G-CSF to to glycosylated G-CSF.	3
25 of 853	Glomerulonephritis and /or persistant hematuria was reported in 25 patients. Of these patients, 13 had no obvious predisposition to renal disease. Renal biopsies were performed in 7 of 13 patients and revealed immune complex glomerulonephritis in six and possible SLE in one.	Severe chronic Neutropenia registry	G-CSF given for years. Improvement in 5 of 10 patients with decreasing or discontinuing G-CSF. But 2 of 3 patients also improved despite continuation of G-CSF without any changes.	4

1	Crescentic glomerulonephritis. IF with IgA and C3 and EM with immune deposits in mesangium c/w IgA nephropathy. Had AKI requiring dialysis	Chronic neutropenia	On chronic G-CSF.<br>Received steroids with improvement of renal function.<br>The patient subsequently had a presentation months later with GI bleed that was recurrent with necrotizing vasculitis in the resected bowel and there was concern for possible pauci-immune etiology, ANCA was negative	5
2	Two patients with SLE and lupus nephritis at baseline.<br>1st patient with class 4/5 lupus nephritis on cellcept who developed acute worsening of renal function and nephrotic proteinuria, hematuria within days of getting G-CSF for neutropenia. Renal biopsy showed class 4 lupus nephritis with crescents and fibrinoid necrosis. Patient ultimately required dialysis.<br>2nd patient with class 4 lupus nephrits with stable creatinine on IV cyclophosphamide. Developed worsening renal function after getting G-CSF for neutropenia. No renal biopsy done. Patient required dialysis.	Neutropenia post treatment for SLE	Both patients had SLE with flare of renal disease after G-CSF<br>Both patients required dialysis	6.
1	AKI requiring dialysis after getting Peg filgrastim, this eventually resolved, patient able to come off dialysis with normalization of creatinine. However, had persistant nephritic sediment and nephrotic range proteinuria.<br>Received repeat cycle of chemotherapy with pegfilgrastim, presented 2 weeks later with severe AKI with creatinine upto 6.7, nephritic sediment and nephrotic proteinuria. Renal biopsy consistant	Neutropenia post chemotherapy for breast cancer	Received Peg filgrastim with chemotherapy.<br>Required dialysis 1st time, 2nd time AKI resolved on its own.<br>No further episodes of AKI when pegfilgrastim was held during subsequent chemotherapy cycles but continued to have glomerular hematuria	7

	with mesangioproliferative GN, IF negative for IgA, there were rare electron dense immune deposits in the glomerular basement membrane and mesangium. AKI resolved on its own.			
1 (child)	Normal baseline urinalysis and renal function. Patient presented about 2 weeks after first dose of filgrastim with hematuria, proteinuria and renal dysfunction. A kidney biopsy few weeks later was consistant with IgA nephropathy, the patient received immunosuppression with steroids and azathioprine with improvement.	Peripheral stem cell donation	Received Filgrastim for 5 days. He received 12.5 mcg/kg/day of filgrastim on day 1 to 3, but 15.6 mcg/kg twice daily on day 4 and once daily on day 5.<br>For the GN, received treatment with steroids and Azathioprine with improvement.	8.

1	At baseline, patient had asymptomatic microscopic hematuria in one of three urine tests, no proteinuria and had normal renal function.<br>On the 5th day of G-CSF she developed macroscopic hematuria, dysmorphic red blood cells in the urine, subsequently developed nephrotic proteinuria, renal function remained normal.<br>Renal biopsy was done about 2 weeks after starting G-CSF and showed segmental endocapillary increase in cellularity with cellular	Peripheral Stem cell donation	Received filgrastim at dose of 5 mcg/kg twice daily for 6 days.<br>Hematuria and proteinuria improved over weeks after discontinuation of G-CSF.<br>Complement, ANA, ANCA, and anti-GBM antibody were normal.<br>Renal function and urine tests were normal at 1 year.	9.

	segmental crescents in 40% glomeruli. IF showed moderately intense IgG in mesangium.			
3	Within days of starting G-CSF, all 3 patients developed visible hematuria, proteinuria; proteinuria nephrotic in two and not quantified in one patient.<br>Two patients had worsening renal function while renal function remained normal in the third patient. One patient had renal biopsy consistant with IgA nephropathy, the other two patients had presumptive diagnosis of IgA nephropathy.	Peripheral Stem cell donation	After stopping G-CSF, there was improvement of renal function noted in the 2 patients with elevated creatinine.<br>There was improvement in proteinuria and microscopic hematuria in 2 patient within weeks but were persistant at 5 months in patient with biopsy proven IgA nephropathy.	10.
1	Development of crescentic GN in renal allograft after receiving filgrastim. This was superimposed on the recurrence of the primary disease (Proliferative GN with monoclonal deposits IgG2λ) in an MPGN pattern in the allograft. Noted to have plasma cell neoplasm with 5% plasma cells. He underwent preparation for autologous stem cell transplant and received filgrastim 480 mcg twice daily for 5 days. This was complicated by worsening creatinine from 2.7 mg/dl to 7.4 mg/dl one day after completing treatment, he became anuric and started dialysis. A repeat allograft biopsy showed crescentic GN with cellular crescents in 72% of the non-sclerotic glomeruli.<br>IF with mesangial and glomerular capillary wall staining for IgG2 ,C3 and λ and electron microscopy showed subendothelial,mesangial and segmentally subepithelial electron dense deposits.	Autologous stem cell transplant in the patient	Patient was anuric requiring dialysis. He received methylprednisolone and four cycles of plasmapheresis. The patient underwent stem cell transplantation six days after allograft biopsy. A repeat allograft biopsy two weeks post stem cell transplant showed no improvement in the GN. The patient remained on hemodialysis as of eight months after the last allograft biopsy. [25]<br>Comparative immunostains the allograft biopsy with crescents and the preceding allograft biopsy showed 13 fold increase in intraglomerular neutrophils and 2.3 fold increase in intraglomerular macrophages in the postfilgrastim biopsy. The authors attributed the crescentic transformation of the patient's known IgG2λ GN to the use of filgrastim	11.

1	Patient with extranodal lymphoma of mucosa associated lymphoid tissue with normal baseline urinalysis and renal function developed renal dysfunction after 3rd cycle of rituximab and bendamustine with pegfilgrastim.<br>He had nephrotic proteinuria and dysmorphic red cells on urine microscopy. C3 was low, rest of serologies were unremarkable.<br><br>Renal biopsy showed necrotizing crescentic glomerulonephritis with mesangial monoclonal IgG λ deposits. The authors proposed that the crescentic GN was related to pegfilgrastim.	Pegfilgrastim given with chemotherapy	The pegfilgrastim was discontinued and he was treated with steroids for 12 weeks with improvement in renal function and proteinuria. However, he continued to have some proteinuria and microscopic hematuria.	12.
1	Patient with known IgA nephropathy At her initial diagnosis of IgA nephropathy, she had presented with proteinuria, estimated glomerular filtration rate of 36 ml/min/1.73 m^2 and was given steroids and achieved remissions.<br>She was in remission at time of the donor evaluation with normal urinalysis and renal function.	Peripheral Stem cell donation	She received G-CSF at 5 mcg/kg dose twice daily for 3 days. She developed visible hematuria on day 3 of mobilization. Her day 4 dose was held, renal function worsened on day of cell harvest but hematuria decreased after holding G-CSF. Due to inadequate cell collection she received 1 more dose of G-CSF after informed consent.<br>Subsequently she had resolution of hematuria and renal function was back to normal by day 8 and urinalysis three months later remained normal.	13.

References

[1] Bonilla MA, Dale D, Zeidler C, et al: Long –term safety of treatment with recombinant human granulocyte colony-stimulating factor (r-metHuG-CSF) in patients with severe congenital neutropenias. Br J Haematol. 1994, 88(4): 723-730.

[2] Sotamatsu M, Kanazawa T, Ogawa C, Watanabe T, Morikawa A: Complication of rapidly progressive glomerulonephritis in severe congenital neutropenia treated with long-term granulocyte colony-stimulating factor. Br J Haematol. 2000, 110 (1): 234-5.

[3] Magen D, Mandel H, Berant M, Ben-Izhak O,Zelikovic I: MPGN type 1 induced by granulocyte colony stimulating factor. Pediatr Nephrol. 2002, 17: 370-372.

[4] Dale DC, Cottle TE, Fier CJ et al: Severe Chronic Neutropenia:Treatment and follow up of patients in the Severe Chronic Neutropenia International Registry. Am J Hematol. 2003, 72 (2): 82-93.

[5] Hill PA: Gastrointestinal small vessel vasculitis in a patient with crescentic glomerulonephritis and longstanding idiopathic neutropenia treated with G-CSF. Pathology. 2003, 35:179-83.

[6] Vasiliu IM, Petri MA, Baer AN: Therapy with Granulocyte colony-stimulating factor in SLE may be associated with severe flares. J Rheumatol, 2006 , 33: 1878-80.

[7 Arora S, Bhargava A, Jasnosz K, Clark B: Relapsing acute kidney injury associated with Pegfilgrastim. Case reports in Nephrology and Urology. 2012, 2 (2): 165-171.

[8] Funokoshi Y, Nazneen A, Nakashima Y, Nakashima K, Okada M, Taguchi T, Moriuchi H: Possible involvement of G-CSF in IgA nephropathy developing in an allogenic peripheral blood SCT donor. Bone Marrow Transplant. 2010, 45(9):1477-8.

[9] Nasilowska-Adamska B, Perkowska-Ptasinka A, Tomaszewska A, Serwacka A, Marianska B: Acute GN in a donor as a side effect of allogenic peripheral blood stem cell mobilization with granulocyte colony-stimulating factor. International Journal of Hematology. 2010, 92: 765-768.

[10] Lee JB, Billen A Lown RN,et al: Exacerbation of IgA nephropathy following G-CSF administration for PBSC collection: suggestions for better donor screening. Bone Marrow Transplantation. 2016, 51: 286-287.

[11]Batal I, Markowitz GS, Wong W, Avasare R,MaparaMY, Appel GB, D'Agati VD: Filgrastim-Induced Crescentic Transformation of Recurrent IgG2λ GN. J Am Soc Nephrol. 2016, 27:1911-1915.

[12] Ebad H, Nayak R, Rashid T, Stokes MB: Acute kidney injury in a 78 year old man with low grade B cell lymphoma. Kidney Int. 2018, 93 (1): 275-276.

[13] Nampoothiri RV, Kumar V, Bharati J, Lad S, Arora K, Malhotra P, Lad D: Hematopoietic stem cell donor with IgA nephropathy: Challenges and management algorithm. Transfusion and Apharesis Science. 2020, 59 (4): 102781.
